# Biochemical characterization of a disease-causing human osteoprotegerin variant

**DOI:** 10.1038/s41598-022-19522-9

**Published:** 2022-09-10

**Authors:** Yin Luo, Miaomiao Li, Ding Xu

**Affiliations:** Department of Oral Biology, School of Dental Medicine, University at Buffalo, The State University of New York, SUNY, Buffalo, NY 14214 USA

**Keywords:** Mechanisms of disease, Proteins

## Abstract

Recently, a human mutation of OPG was identified to be associated with familial forms of osteoarthritis. This missense mutation (c.1205A =  > T; p.Stop402Leu) occurs on the stop codon of OPG, which results in a 19-residue appendage to the C-terminus (OPG^+19^). The biochemical consequence of this unusual sequence alteration remains unknown. Here we expressed OPG^+19^ in 293 cells and the mutant OPG was purified to homogeneity by heparin affinity chromatography and size exclusion chromatography. We found that in sharp contrast to wildtype OPG, which mainly exists in dimeric form, OPG^+19^ had a strong tendency to form higher-order oligomers. To our surprise, the hyper-oligomerization of OPG^+19^ had no impact on how it binds cell surface heparan sulfate, how it inhibits RANKL-induced osteoclastogenesis and TRAIL-induced chondrocytes apoptosis. Our data suggest that in biological contexts where OPG is known to play a role, OPG^+19^ functions equivalently as wildtype OPG. The disease-causing mechanism of OPG^+19^ likely involves an unknown function of OPG in cartilage homeostasis and mineralization. By demonstrating the biochemical nature of this disease-causing OPG mutant, our study will likely help elucidating the biological roles of OPG in cartilage biology.

## Introduction

Osteoprotegerin (OPG) is a subject of extensive study due to its critical role in regulating osteoclastogenesis and bone remodeling. As a soluble decoy receptor, OPG inhibits osteoclastogenesis by competing with the receptor activator of the nuclear factor kappa-B (RANK), expressed on the membrane of preosteoclasts, for the binding of the nuclear factor kappa-B ligand (RANKL). When OPG is present, it limits the bioavailability of RANKL to RANK and effectively inhibits osteoclastogenesis^1,2^. The role of OPG as a master negative regulator of osteoclastogenesis is manifested by the profound osteoporotic phenotype displayed by *opg* null mice, which stems from the uncontrolled osteoclastogenesis and bone resorption^3,4^. Recently, we and others have also shown that heparan sulfate (HS) is an essential regulator of OPG activity^5–7^. When the interaction between OPG and osteoblast HS was disrupted, OPG was not able to inhibit RANKL efficiently, which resulted in osteoporosis in murine models.

Compared to the well-studied role of OPG in bone remodeling, the function of OPG in cartilage is less clear. Abundant OPG expression has been observed in both human and murine chondrocytes^8,9^, and several lines of evidence from animal studies pointed to a direct role of OPG in maintaining cartilage health. Several groups have reported that *Opg* null mice displayed osteoarthritis (OA) phenotype with severe degenerative multiple joint disease, thinner cartilage layers and progressive loss of cartilage matrix at young age^10,11^. Importantly, even heterozygous *Opg* null mice display OA phenotype when they are aged, which suggests that a partial deficiency in OPG was enough to trigger cartilage degeneration^10^. In a murine model of induced OA, it was demonstrated that OPG expression in the articular cartilage was significantly reduced after an injury, and intraarticular injection of OPG protected the articular cartilage from the degeneration^10,12^.

Chondrocytes express two different ligands for OPG. RANKL appears to be widely expressed by chondrocytes^9,13^, but whether it plays a direct role in cartilage homeostasis remains unclear. Chondrocytes also expresses tumor necrosis factor-related apoptosis-inducing ligand (TRAIL)^10,12,14^. Like RANKL, TRAIL also belongs to the TNF superfamily and is a potent inducer of apoptosis by binding to cell surface TRAIL receptors^15^. While the expression of TRAIL in healthy cartilage is minimal, its expression is highly upregulated in cartilage with OA^10,12^. Because apoptosis of chondrocytes plays a crucial role in cartilage degeneration^16–18^, it was hypothesized that OPG protects cartilage by preventing TRAIL induced chondrocyte apoptosis^10,12^.

Recently, a human mutation of OPG was described to be associated with an early-onset familial form of OA. Interestingly, patients carrying this mutation show deposition of calcium pyrophosphate crystals in the cartilage structure, which is the clinical characteristic of calcium pyrophosphate deposition disease (CPDD)^19,20^. This discovery has shed new light on the role of OPG in cartilage biology because it suggests that OPG can regulate cartilage health with more than one mechanism. This missense mutation (c.1205A > T; p.Stop402Leu) occurs on the stop codon of OPG (TNFSRF11B), resulting in a mutant with 19 additional amino acids at the C-terminus^19,20^. Interestingly, patients from all three families carry the mutation in a heterozygous background, which suggests that this mutation causes OA/CPPD in an autosomal dominant manner. However, the molecular basis by which this mutation causes OA/CPPD remains unknown.

In this report we sought to understand the biochemical and biological consequence of this OPG mutation. We found that the recombinant OPG^+19^ existed almost exclusively as higher order oligomers. Unexpectedly, the oligomerization did not have an obvious impact on OPG-HS interaction on osteoblast surface. The oligomerization per se also had little effect on the inhibitory activity of OPG^+19^ towards RANKL in osteoclastogenesis assays. In addition, the mutant OPG displayed normal inhibitory activity towards TRAIL-induced chondrocytes apoptosis. Our data suggests that the mechanism of OPG^+19^ causing the disease likely involves an unknown function of OPG in cartilage homeostasis and mineralization.

## Results

### Multiple OPG stop codon mutations were found in different ethnic groups

In addition to the three families that have been reported to carry the c.1205A > T; p.Stop402Leu mutation^19,20^, we were curious whether additional carriers can be identified in large scale genomic sequencing dataset that is now publicly available. In gnomAD database^21^, we did not find any additional case of c.1205A > T; p.Stop402Leu mutation. However, to our surprise we found two additional OPG stop codon missense mutations. These mutations are 1204 T > C, which would change the stop codon to glutamine; and 1206A > C, which would change the stop codon to tyrosine (Table I). These mutations will lead to changes in protein sequence that are highly similar to the mutation investigated in this study, both of which would also generate 19 amino acids appendages that differ from OPG^+19^ mutant only at position 402 (Gln and Tyr, respectively, instead of Leu). All individuals identified in the database are heterozygous carriers of these mutations. Interestingly, the 1204 T > C mutation was only found in European population with a moderate frequency of 1 in 16,570 individuals, while the 1206A > C was found in South Asian population with a frequency of 1 in 17,254 individuals (Table I). It remains unknown whether carriers of these mutations would manifest OA/CPPD that is similar to OPG^+19^ carriers. Our finding suggest that stop codon missense mutations of OPG can be found in different ethnic background in moderate numbers.

**Table I:** Additional stop codon mutations of human OPG (TNFRSF11B).

	Mutation 1 (this study)	Mutation 2	Mutation 3
Protein change	Stop402Leu	Stop402Gln	Stop402Tyr
Transcript change	1205A > T [T**A**A] > [T**T**A]	1204 T > C [**T**AA] > [**C**AA]	1206A > C [TA**A**] > [TA**C**]
Genomic location (GRCh38.p13)	chr8:118,924,375	chr8:118,924,376	chr8:118,924,374
SNP identifier	N/A	rs774713244	rs1381214863
Frequency (gnomAD v2.1.1 and V3.1.2)	Not found	1 : 39,760 (5/198,800)	1: 198,800
Ethnicity	3 families reported. (2 Jewish, 1 not disclosed)	All European 1:16,570 (5/82,851)	South Asia 1/17,254

### Purification and characterization of OPG^+19^ mutant

**Figure 1:**
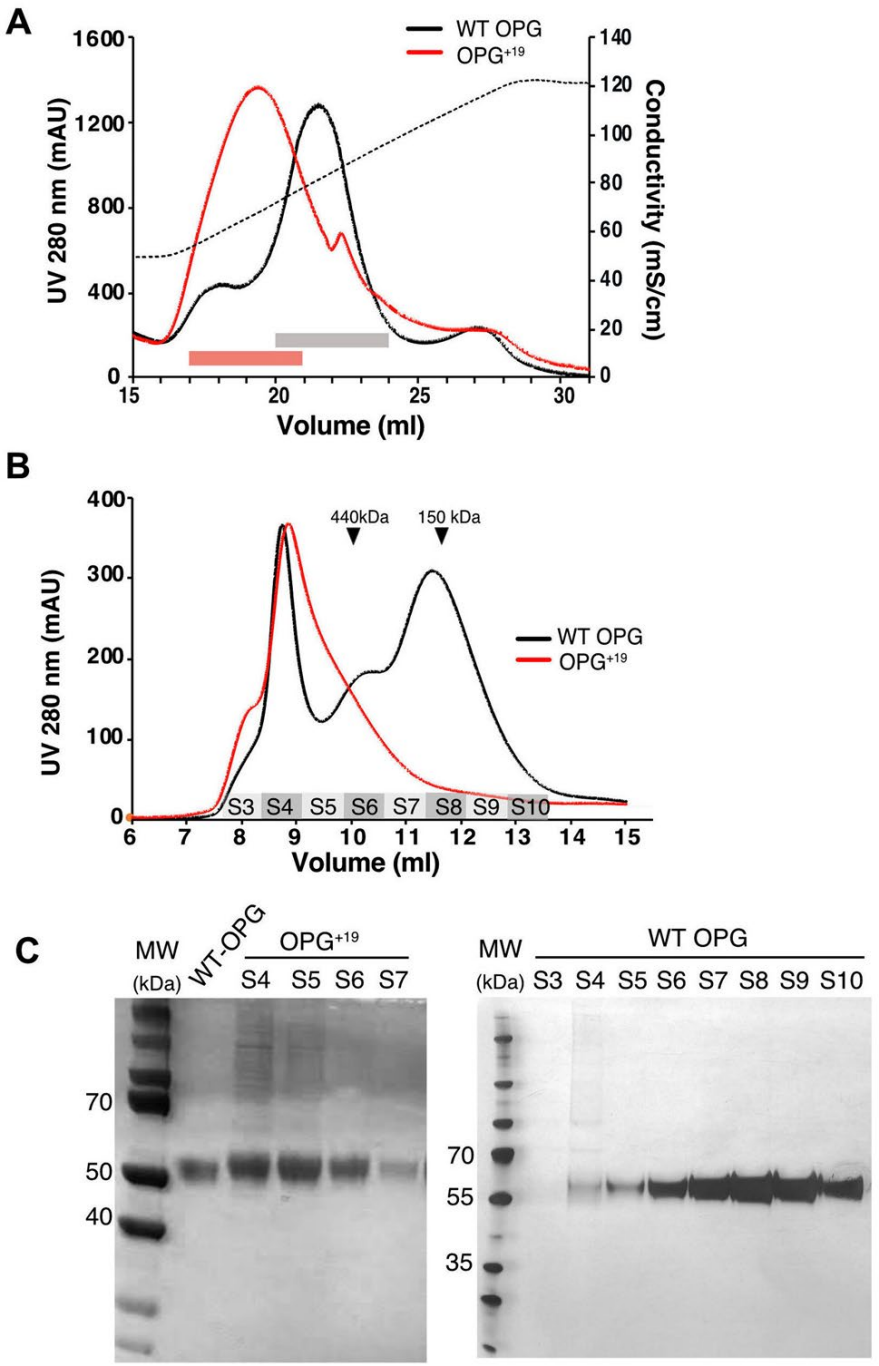
Purification of wild-type and mutant human OPG. (**A**) Binding of hOPG to heparin Sepharose column. The dotted line represents the salt gradient (in conductivity mS/cm). WT OPG was eluted as two peaks (low-salt peak at 18 ml is monomer, high-salt peak at 21.5 ml is dimer), whereas mutant hOPG was eluted as a single broad peak. The peak fractions of mutant (red box) and WT OPG dimer (gray box) were further purified by size-exclusion chromatography. (**B**) Purification of OPG on Superdex200 column. Fraction numbers are labeled at the bottom of the chromatogram (S3-S10). Elution positions of ferritin (440 kDa) and IgG (150 kDa) are marked with black triangles. (**C**) SDS-PAGE analysis of the Superdex200 peak fractions of OPG^+19^ (S4-S7) and WT OPG (S3-S10). All samples were reduced with beta-mercaptoethanol (2-Me). The earlier peak of WT OPG (fraction S4) contains multiple secreted HS-binding proteins co-purified with OPG on heparin Sepharose column.

Our previous studies on OPG-HS interaction has identified that the HS-binding site of OPG is located close to the C-terminus^6^. Because the OPG^+19^ mutant added an additional 19 amino acid to the C-terminus, we hypothesized that these additional residues might interfere with the HS-binding site. Using our established expression system of murine OPG, both wildtype human OPG (WT OPG) and OPG^+19^ mutant were expressed successfully in 293-freetyle cells. When culture supernatants containing WT OPG or OPG^+19^ were applied onto heparin Sepharose column, we found that their elution profiles were drastically different. Like what we observed with murine OPG^6^, WT OPG showed two distinct peaks when eluted by salt gradient from heparin column (Fig. 1A). The low-salt small peak (at 18 ml) represents monomeric OPG, while the high-salt dominant peak (at 21.5 ml) represents dimeric OPG. In contrast, OPG^+19^ was eluted from heparin column as a broad single peak, and the peak position was midway between the elution positions of monomeric and dimeric WT OPG. To determine the molecular weight (MW) of OPG^+19^, the peak fractions from the heparin column were combined and analyzed by size exclusion chromatography (SEC). Compared to WT OPG dimer, which was eluted at 11.4 ml, OPG^+19^ was eluted much earlier with a peak at 8.9 ml (Fig. 1B). The SEC profile indicates that OPG^+19^ exists as large oligomers with MW > 400 kDa. To verify the purity of OPG^+19^, the peak SEC fractions (S4-S7, Fig. 1C, left panel) were reduced with 2-mercaptoethanol and analyzed by SDS-PAGE. Indeed, these fractions all contains OPG^+19^ with a purity > 90%. Of note, although the SEC profile of WT OPG also contains a peak at 8.9 ml (Fig. 1B), SDS-PAGE analysis of the SEC fractions clearly showed that the peak (fraction S4) contains a mixture of co-purified HS-binding proteins (Fig. 1C, right panel). This is in sharp contrast to S4 fraction of OPG^+19^, which contains an intense band of OPG in addition to the co-purified high MW HS-binding proteins (Fig. 1C, left panel).

### OPG^+19^ exists in multiple higher-order oligomeric forms

**Figure 2:**
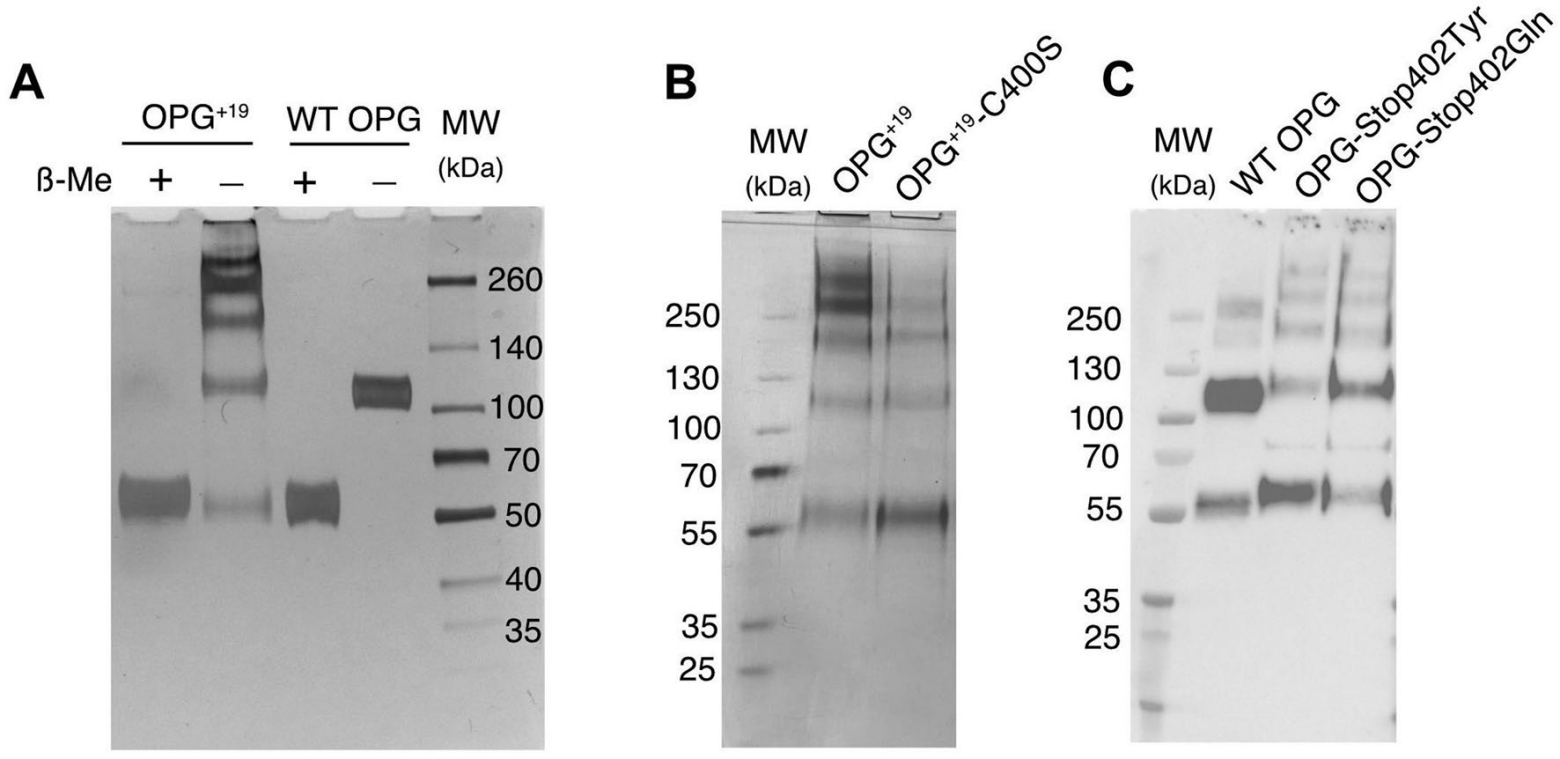
OPG^+19^ tend to form large oligomeric complexes. (**A**) SEC purified WT dimer (fraction S7-S10 from Fig. 1C) and OPG^+19^ (fraction S5 from Fig. 1C) were resolved by SDS-PAGE and visualized by silver staining. Samples were run either under non-reducing condition to preserve intermolecular disulfide bound, or reduced with 2-Me. (**B**) SEC purified OPG^+19^ and OPG^+19^/Cys400Ser compound mutant (OPG^+19^-C400S) were visualized by silver staining under non-reducing condition. (**C**) 293F cells were transfected with WT OPG, OPG-Stop402Tyr or OPG-Stop402Gln mutants, and the culture medium were harvested 3 days post transfection and resolved by SDS-PAGE. The gel was blotted with rabbit anti-OPG polyclonal antibody.

To better understand the biochemical nature of the oligomeric OPG^+19^, purified OPG^+19^ (S5 fraction from Fig. 1C) and WT OPG dimer were visualized by SDS-PAGE under both reducing and non-reducing conditions. As expected, WT OPG migrated at 55 and 110 kDa under reducing and non-reducing condition, respectively (Fig. 2), which is consistent with that it exists as a disulfide-linked dimer. In contrast, while under reducing condition OPG^+19^ also migrated around 55 kDa, the unreduced OPG^+19^ mainly migrated as multiple high MW bands including dimer, tetramer, hexamer and octamer. The fact that all higher order oligomers collapsed into monomer after reduction suggests that intermolecular disulfide bond (through Cys-400) likely plays an essential role in assembling the oligomers. In addition, because a large portion of the oligomers were resistant to SDS and heat denaturing, it suggests that the additional C-terminal peptide (^402^LLEMAIELFPHNWRDPMDE^420^) was likely involved in intermolecular hydrophobic interactions due to a high content of hydrophobic residues (11 out of 19). To test whether the C-terminal Cys-400 directly involves in assembling the oligomer, we have prepared a variant of OPG^+19^ mutant by introducing Cys-400-Ser mutation. As expected, OPG^+19^ /C400S mutant displayed greatly reduced tendency to form higher order oligomers (Fig. 2B), which suggests that Cys400 indeed plays a role in assembling the higher-order oligomers. At last, we have also overexpressed the other two OPG stop codon mutants (OPG-Stop402Gln and OPG-Stop402Tyr) in 293F cells and found they also had strong tendency to form higher order oligomers (Fig. 2C). This finding implies that carriers of these two mutations might also manifest CPPD.

### OPG^+19^ retains full capacity to bind cell surface HS

**Figure 3:**
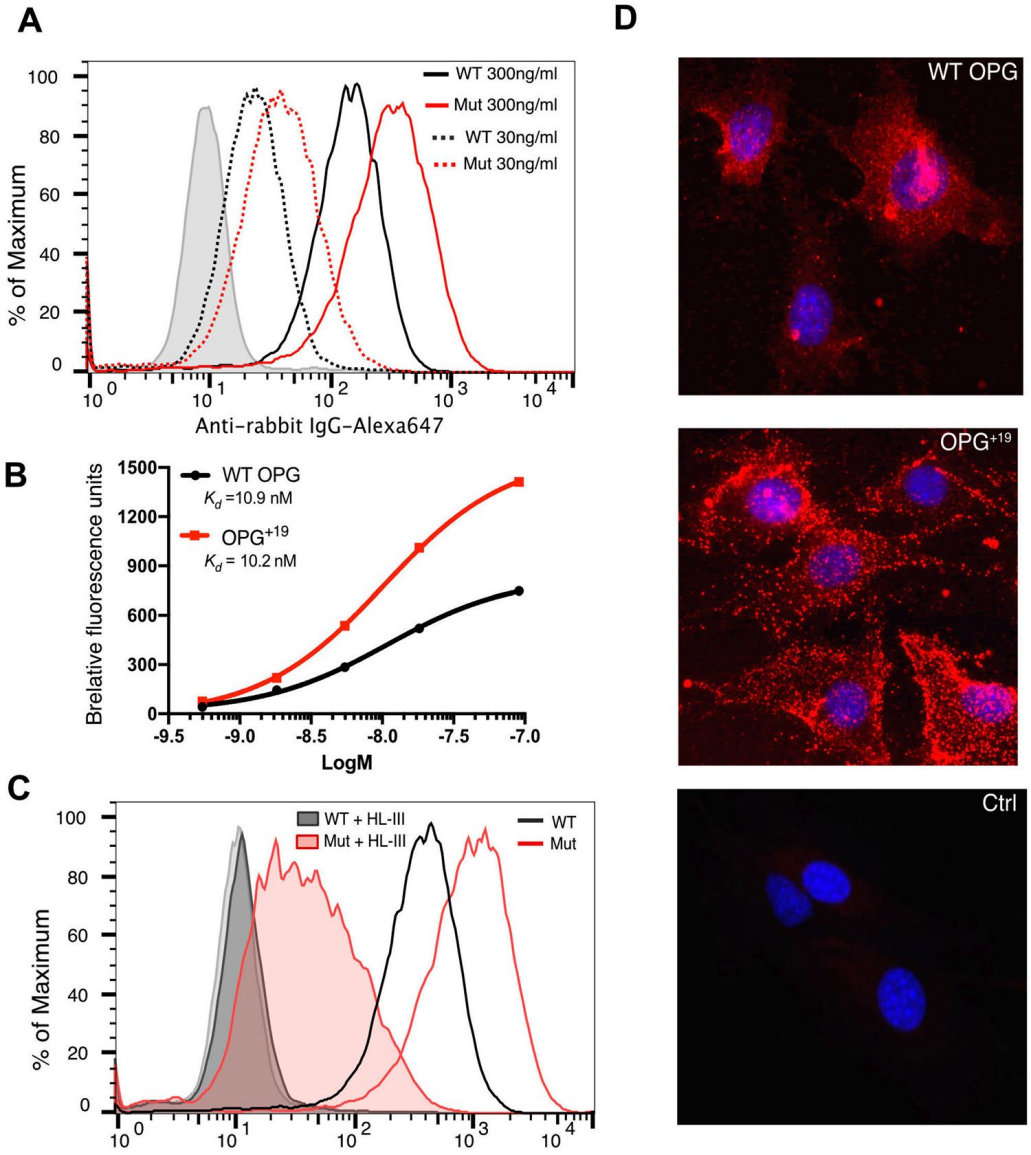
OPG^+19^ preserves HS-binding capacity. (**A**) Binding of WT and mutant human OPG (30 ng/ml and 300 ng/ml) to MC3T3 cells were determined by a FACS-based binding assay. The bound OPG were detected by staining with a rabbit anti-OPG antibody, followed by anti-rabbit-IgG–Alexa647. The shaded histogram is from cells stained only with primary and secondary antibodies. (**B**) Apparent binding affinity of WT OPG and OPG^+19^ to MC3T3 cells was determined by FACS. Concentration of OPG tested were between 30 ng/ml and 5 µg/ml. (**C**) Binding of WT and mutant OPG (100 ng/ml) to untreated MC3T3 cells or cells pretreated with heparin lyase III (HL-III, shaded histogram) (**D**) OPG^+19^ display a unique binding pattern to osteoblast surface HS. MC3T3 cells were incubated with either WT or mutant OPG, both at 300 ng/ml. Bound OPG were detected with 0.1 μg/ml rabbit anti-OPG, followed by goat anti-rabbit-594. Magnification = 200×.

Studies from our lab has shown that binding of OPG to HS plays an essential role in promoting the inhibitory activity of OPG towards RANKL^5^. Based on the reduced binding of oligomeric OPG^+19^ to heparin column, we reasoned that OPG^+19^ would also display diminished binding to cell surface HS, which would likely be a disease-causing mechanism of OPG^+19^. However, contrary to our prediction, osteoblasts incubated with OPG^+19^ gave a binding signal that is twofold higher than cells incubated with WT OPG (Fig. 3A). Performing this binding experiment with different concentrations of OPG allowed us to calculate the apparent binding affinities of WT OPG and OPG^+19^ to cell surface. Interestingly, despite the higher binding signal given by OPG^+19^ , the apparent binding affinity of OPG^+19^ was identical to that of WT OPG, both at ~ 10 nM (Fig. 3B). To determine whether binding of OPG^+19^ to cell surface was HS-dependent, we further performed binding experiments on cells pretreated with heparin lyase III (HL-III), which removes cell surface HS. As we previously reported^6^, binding of WT OPG to osteoblasts was almost entirely HS-dependent, with less than 0.5% binding remained after HL-III treatment (Fig. 3C). In contrast, although binding of OPG^+19^ was also predominantly mediated by cell surface HS, after HL-III treatment a significant residual binding (5%) remained (Fig. 3C). This result suggests that OPG^+19^ can bind additional cell surface molecules other than HS, whereas WT OPG only binds HS at cell surface.

### OPG^+19^ displays a unique binding pattern to HS

To visualize how OPG^+19^ binds to cell surface HS, we performed immunostaining of osteoblasts with bound OPG^+19^ and WT OPG. Interestingly, while WT hOPG bound HS rather evenly with a fine grainy staining pattern, OPG^+19^ bound HS in a sparse grainy pattern with very large bright dots (Fig. 3D). The size of the OPG^+19^ signal likely reflects the size of the oligomer, which is much bigger than WT OPG dimer. This unique binding pattern of OPG^+19^ to cell surface HS likely might contribute to the discrepancy in its binding behaviors to heparin Sepharose column and cell surface HS. Compared to cell surface HS, immobilized heparin on Sepharose beads is shorter and less flexible, which might have difficulty to fully occupy all available HS-binding sites on large OPG^+19^ oligomer. As a result, OPG^+19^ oligomer would have less interactions with heparin on a per monomer basis compared to WT OPG dimer, which is fully occupied by heparin.

### The anti-RANKL activity of OPG^+19^ was largely normal in osteoclastogenesis assays

**Figure 4:**
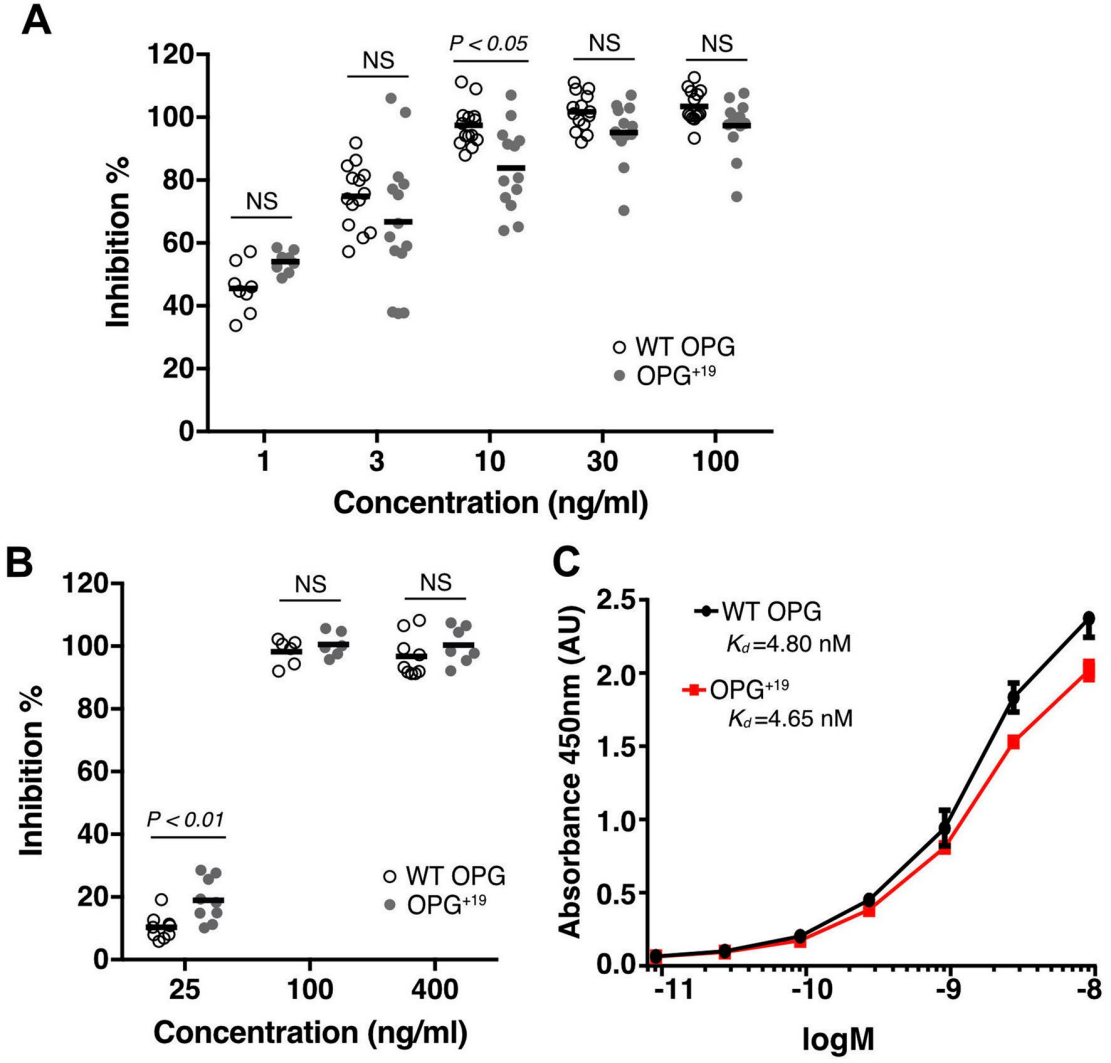
OPG^+19^ has largely normal anti-RANKL activity in osteoclastogenesis. (**A**) Inhibition of osteoclastogenesis in osteoblasts/bone marrow macrophage co-culture by WT hOPG or mutant hOPG at 1, 3, 10, 30, 100 ng/ml. The extent of osteoclastogenesis is assessed by an enzymatic assay of TRAP activity in whole cell lysate. (**B**) Inhibition of osteoclastogenesis in bone marrow macrophage monoculture by WT hOPG or mutant hOPG at 25, 100 and 400 ng/ml. Osteoclastogenesis is induced by recombinant soluble RANKL (50 ng/ml) and M-CSF (20 ng/ml). (**C**) Binding affinity of WT hOPG and mutant hOPG to immobilized RANKL was determined by enzyme-linked immunosorbent assay (ELISA).

To examine whether the inhibitory activity of OPG^+19^ towards RANKL was altered, we performed monoculture and co-culture osteoclastogenesis assays. In the co-culture assay, primary murine osteoblasts were co-cultured with bone marrow macrophage (BMM) and the osteoclastogenesis of BMM was induced by osteoblasts expressed RANKL. As reported previously^6^, inhibition of osteoclastogenesis by OPG was highly robust in this model, requiring only 1 ng/ml of OPG to reach 50% of inhibition (Fig. 4A). We found that OPG^+19^ displayed potent inhibition of osteoclastogenesis, an effect that was indistinguishable from WT OPG in all concentrations tested except at 10 ng/ml. Although at this concentration WT OPG did display higher inhibition efficiency (statistically significant), the difference was quite small (95% vs 83%). In monoculture assay, osteoclastogenesis was driven by exogenously added soluble RANKL (50 ng/ml), and adding OPG dose-dependently inhibits the extent of osteoclastogenesis. We found that dose response of the WT OPG and OPG^+19^ was almost identical (Fig. 4B), and at the lowest concentration OPG^+19^ even performed a little better than WT OPG. This result suggests that the inhibitory activity of OPG^+19^ towards RANKL was intact. Indeed, we further performed binding assay of OPG to immobilized RANKL and found the binding affinities of WT OPG and OPG^+19^ to RANKL were almost equal (Fig. 4C). Combined, these data demonstrated that OPG^+19^ has no inherent defect in inhibiting both membrane-attached (in co-culture) and soluble (in monoculture) RANKL.

### The anti-TRAIL activity of OPG^+19^ was intact in chondrocyte apoptosis assay

**Figure 5:**
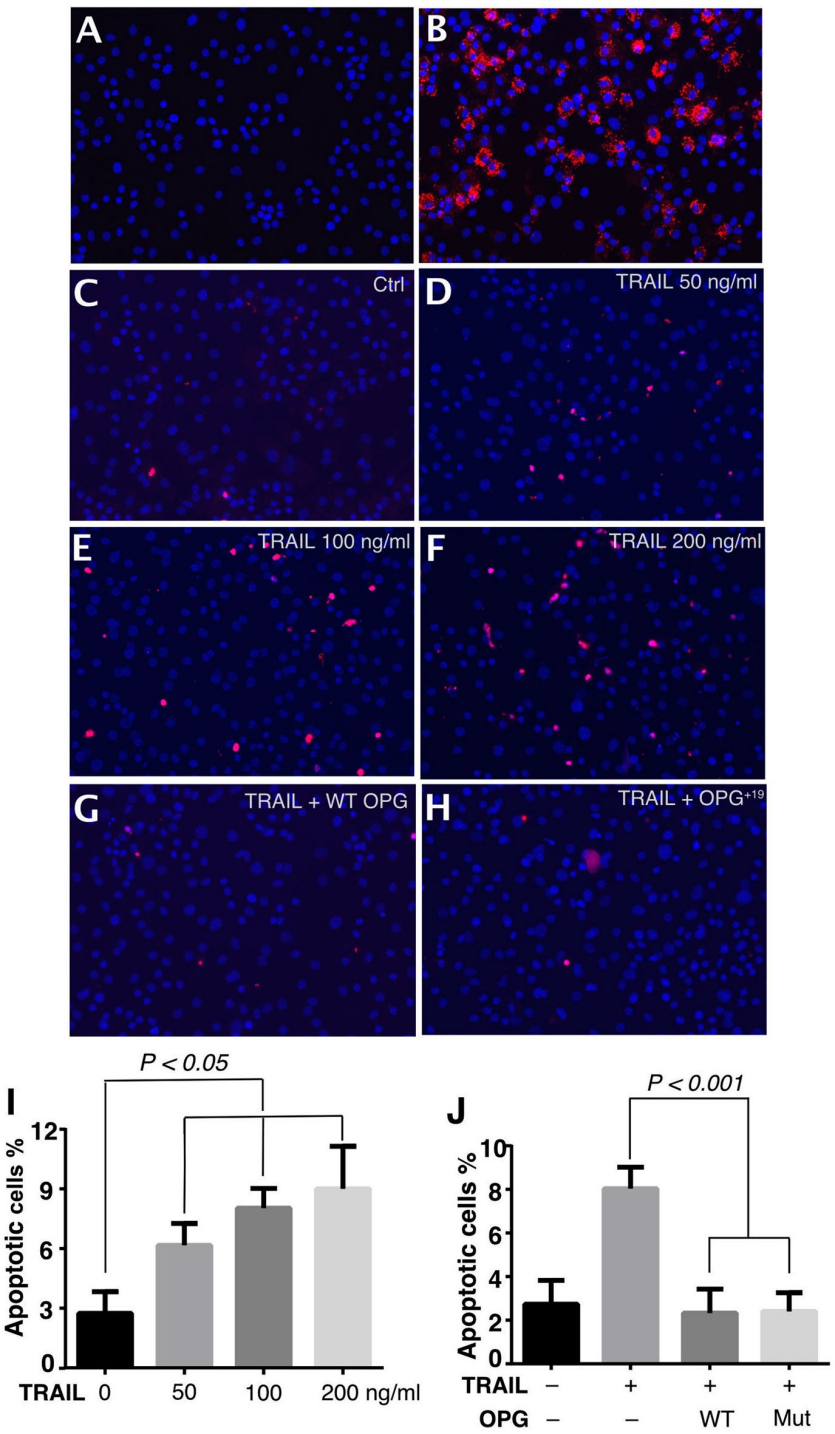
Both WT and mutant OPG inhibit TRAIL-induced chondrocytes apoptosis. (**A**) Primary murine articular chondrocytes were confirmed by Collagen II staining with a mouse anti-Collagen II monoclonal antibody, followed by anti-mouse Alexa594. (**B**) Chondrocyte were incubated with control mouse IgG2, followed by anti-mouse Alexa594. (**C–F**) TUNEL staining of untreated chondrocytes (**C**), or chondrocytes treated with 50 (**D**), 100 (**E**) and 200 ng/ml (**F**) of TRAIL. Apoptotic cells were stained red. Nuclei were stained blue with DAPI. (**G–H**) TUNEL staining of chondrocytes co-treated with 100 ng/ml TRAIL and 400 ng/ml WT (**G**) or mutant hOPG (**H**). (**I**) Quantification of the extent of chondrocytes apoptosis induced by TRAIL. (**J**) Quantification of chondrocytes apoptosis when co-treated with TRAIL and WT or mutant hOPG.

While TRAIL-induced chondrocytes apoptosis has been suggested to play a role in cartilage degeneration in osteoarthritis^10,12,14^, no study has definitively shown that OPG inhibits TRAIL-induced apoptosis in chondrocytes. Here by using primary murine articular chondrocytes (Fig. 5A, B shown chondrocytes actively secrete type-II collagen), we show that TRAIL dose-dependently induced apoptosis of articular chondrocytes (Fig. 5C–F and I). When WT OPG or OPG^+19^ was added into the culture along with TRAIL, we found the apoptosis was suppressed by both forms to similar extent (Fig. 5G, H , I, J). This data demonstrated that TRAIL is indeed a potent inducer of chondrocyte apoptosis, and that both WT OPG and OPG^+19^ can effectively inhibit TRAIL-induced apoptosis.

### OPG^+19^ overexpressed by human chondrocytes also exists in hyper-oligomerized forms.

**Figure 6:**
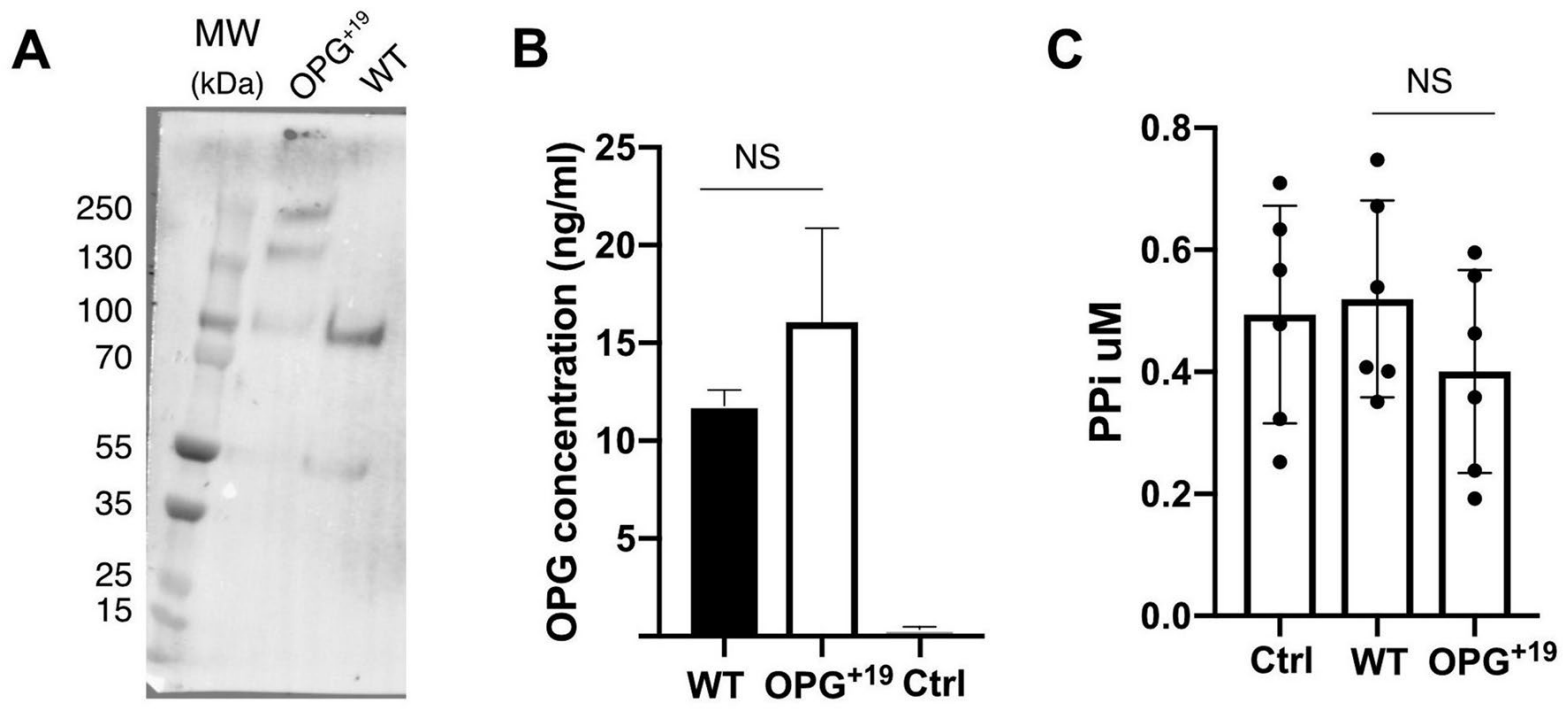
OPG^+19^ overexpressed by human chondrocytes also exists in hyper-oligomerized forms. (A-B) Expression of WT OPG and OPG mutant in a human chondrocyte cell line. Proteins were analyzed 2 days post transfection. (**A**) Culture medium were harvested and OPG purified by heparin Sepharose column. Purified WT and mutant OPG were resolved by SDS-PAGE and blotted with rabbit anti-OPG polyclonal antibody. (**B**) Concentration of secreted WT and mutant OPG in transfected chondrocyte culture medium. OPG concentration was determined by ELISA. Ctrl: medium from untransfected chondrocyte culture. n = 5. (**C**) The PPi concentration in the culture medium were determined 2-days post transfection using a commercial PPi assay kit. Ctrl: medium from untransfected chondrocyte culture. n = 6.

To examine whether OPG^+19^ also exists in hyper-oligomerized form when expressed by chondrocytes, a dominant cell type in cartilage, we overexpressed OPG^+19^ in a human chondrocyte cell line (TC28a2). Similar to 293 cells, chondrocytes expressed OPG^+19^ also exists in a higher-order oligomers (Fig. 6A), and the expression level of OPG^+19^ was also comparable to WT OPG (Fig. 6B). Using the same overexpression system, we further examined whether OPG^+19^ has an impact on secretion of pyrophosphate (PPi) in chondrocytes because enhanced secretion of PPi has been shown to be associated of CPDD. However, we found overexpression of either WT OPG or OPG^+19^ did not alter extracellular PPi concentration compared to untransfected TC28a2 cells (Fig. 6C).

## Discussion

Since its identification in late 1990s as a major negative regulator of bone resorption^2^, OPG has occupied a prominent position in the field of bone remodeling. In-depth understanding of the working mechanism of OPG as a natural inhibitor of RANKL has led to development of Denosumab^22^, a now widely prescribed anti-RANKL mAb for osteoporosis, which essentially performs the exact function of OPG. As an essential regulator of bone remodeling, loss-of-function mutations of OPG cause a severe genetic bone disease called Juvenile Paget’s Disease (JPD)^23^. The affected patients display progressive bone deformity as a result of abnormal bone turnover, reflecting the critical roles of OPG in bone homeostasis. Close to 10 mutations of OPG have been identified to cause JPD, and interestingly, the disease severity appears to be associated with the extend of deactivation of the OPG function^23^. Severe loss-of-function mutations of OPG, which directly affects RANKL binding, often led to death in early adulthood. In contrast, patients with mutations that preserve RANKL binding but affects OPG dimerization and HS-binding, displayed slower progression of JPD phenotypes^24–26^.

In 2015 Ramos et al. first reported a novel OPG mutation that was associated with familial forms of OA^20^. In contrast to OPG mutations in JPD, which causes disease in an autosomal recessive fashion, this new OPG mutation (c.1205A > T; p.Stop402Leu) causes OA in an autosomal dominant fashion. The nature of the mutation is also unique in that 19 additional amino acids were appended to the C-terminus of OPG due to the missense mutation of the stop codon. In this report we also identified additional OPG stop codon missense mutations (c.1204 T > C; p.Stop402Gln; and c.1206A =  > C; p.Stop402Tyr) from gnomAD database, with a frequency of around 1: 40,000 and 1:200,000, respectively, across all ethnic groups. It is curious that mutations have occurred on all three positions of the stop codon, and importantly, all three mutations would result in highly similar 19 amino acids appendages. Based on the current sequencing data, if all OPG stop codon mutations were lumped together, they would likely form a group with an occurrence rate of less than 1:30,000 individuals across all population. To understand the disease-causing mechanisms of the OPG^+19^ mutation, the two outstanding questions we need to address are: (1) how these 19 additional residues change the biochemical properties of OPG; and (2) how these biochemical changes cause OA in a dominant manner.

Studies from our lab and others have shown clearly that the N-terminal Cysteine Rich Domains (CRDs) are responsible for ligands (RANKL and TRAIL) binding, while the three C-terminal domains are responsible for HS-binding and dimerization^6,27^. Because the majority of the HS-binding residues are located only 20–40 residues away from the C-terminus, and several critical residues involved in OPG dimerization are located even closer to the C-terminus (C400, F385 and L386)^28^, we hypothesized that the OPG^+19^ mutation might alter how the mutant dimerizes and/or interacts with HS. Indeed, our analysis of purified OPG^+19^ mutant has shown clearly that the additional 19 residues rendered the mutant a strong propensity to hyper-oligomerize. Based on our SEC analysis, the OPG^+19^ mostly existed in octamers to dodecamers, while a smaller portion of hexamer and tetramer were also present. Somewhat unexpectedly, while the hyper-oligomerization had a negative impact on OPG^+19^ binding to heparin column, it did not alter the capacity of OPG^+19^ to bind osteoblast cell surface HS (Fig. 3). In sum, our biochemical analysis pointed to hyper-oligomerization, rather than HS-binding, as the most plausible culprit for the disease-causing mechanism.

Interestingly, we discovered that hyper-oligomerization of OPG^+19^ did not have a negative effect on its RANKL-binding affinity and how it inhibits osteoclastogenesis in both monoculture and co-culture models (Fig. 4). Our result confirmed that the hyper-oligomerization state is compatible with normal access of RANKL to the N-terminal CRDs of OPG. Of note, a recent study of the OPG^+19^ mutant suggested that this mutant was less effective than WT OPG in inhibition of osteoclastogenesis^29^. However, in contrast to our study, in that study the OPG was never fully purified, which undoubtedly lowers the confidence in accurate determination of protein concentration, especially when the mutant exists in large oligomers. Furthermore, we also found that anti-TRAIL activity of OPG^+19^ was identical to that of WT OPG in a chondrocyte apoptosis model, which again suggests normal accessibility of the N-terminal CRD domains. Thus, in two biological contexts where OPG has known functions, our study shown that OPG^+19^ has normal biological activities that is indistinguishable from WT OPG.

Based on our biochemical and activity analysis of the OPG^+19^, it is clear that much more work need to be done to reveal the true disease-causing mechanism of this mutant. The difficulty lies in the fact that we do not fully understand the role of OPG in cartilage biology, and in particular the connection between OPG and calcium pyrophosphate crystal deposition in cartilage. While our study does not support OPG^+19^ being either a loss-of-function or gain-of-function mutation, we predict that the mutation is more likely a gain-of-function for the following two reasons. First, many loss-of-function mutations of OPG are known, but none of those has been associated with CPPD. If enhanced calcium pyrophosphate crystal deposition is associated simply with reduced OPG activity, one would expect to find severe CPPD manifestation in patients with JPD (where patients have severely reduced OPG expression/activity), which was not the case. Second, from a protein–protein interaction point of view, OPG^+19^ is predisposed to engage in some novel interaction partners. In this regard, several recent papers reported that even single missense mutation is sufficient to engage novel protein–protein interactions and causing genetic diseases through gain-of-function mechanisms^30,31^. The two novel biochemical properties of OPG^+19^—forming higher order oligomers and gaining 19 additional residues—in principle can both contribute to engaging novel protein interactions. Indeed, in our cell surface binding experiment, it is clear that OPG^+19^ can bind to other cell surface molecules in addition to HS (Fig. 3C). In sum, it appears that the key to solve the mysterious disease-causing mechanism of OPG^+19^ lies on identifying its novel interacting partners, which will likely reveal novel insights of cartilage homeostasis and mineralization.

## Materials and methods

### Expression and purification of recombinant human OPG

Recombinant full-length human OPG was produced in 293-freestyle cells (ThermoFisher Scientific). The complete open reading frame of full-length human OPG was synthesized and cloned into pcDNA3.4-TOPO vector (ThermoFisher). Stop codon mutations (Stop402Leu, Stop402Gln, Stop402Tyr and Stop402Leu-Cys400Ser) was generated by site-directed mutagenesis and the mutation was confirmed by Sanger sequencing. Transient transfection was performed using FectoPro transfection reagent (Polyplus transfection). For 100 ml culture, 293F cells were transfected at 1 × 10^6^/ml with 50 µg of expression vector and 80 µl FectoPro transfection reagent. Expression was allowed for 5 days at 31 °C, by which time the medium were harvested for protein purification.

Purification of hOPG from medium was carried out using HiTrap heparin-Sepharose column (with Buffer A, 25 mM HEPES, pH7.1, 150 mM NaCl; and Buffer B, 25 mM HEPES, pH7.1, 2 M NaCl) and Superdex 200 column (GE Healthcare) (with 25 mM HEPES, pH7.1, 150 mM NaCl as running buffer) as previously described^6^. Both WT and mutant OPG were purified to > 95% purity as examined by silver staining. All binding and functional assays were performed with SEC purified WT OPG dimer, and SEC purified high MW oligomers of OPG^+19^ (fraction S5 and S6 of Fig. 1C). After purification by SEC column, the purified proteins were flash freeze with liquid nitrogen and stored at − 80 °C freezer.

### Fluorescence-Activated Cell Sorting

MC3T3-E1 cells were lifted from culture dish using Accutase (Biolegend) and incubated with WT or mutant hOPG at different concentrations (30–5000 ng/mL) in PBS with 0.1% BSA for 1 h at 4 °C. Bound hOPG was stained with our rabbit anti-mouse/human OPG (1 µg/mL) for 1 h at 4 °C, followed by goat anti-rabbit IgG-Alexa 647 (1:1,000; ThermoFisher Scientific) for 30 min and analyzed by flow cytometry. In some experiments, cells were pretreated with recombinant heparin lyases III (5 milliunits/ml, produced in house as a recombinant protein in E. coli) for 15 min at room temperature prior to binding experiments.

The rabbit anti-mouse/human OPG antibody was developed by immunizing rabbit with full-length murine OPG, and it shows strong cross-reactivity with human OPG. The antibody was affinity purified from the anti-serum with a column immobilized with recombinant murine OPG.

### Immunostaining

MC3T3-E1 cells were cultured on cell culture chambered coverslips. Cells were incubated with WT or mutant hOPG (300 ng/ml) for 1 h at 4 °C and fixed with 4% PFA. Immunofluorescence staining was performed using 0.3 µg/ml rabbit anti-OPG antibody, followed by goat-anti-rabbit-594 (ThermoFisher). Images were taken with a Nikon Ci-S fluorescence microscope and merged using Image J software.

### Osteoclastogenesis assay

*Co-culture Osteoclastogenesis Assay*—Primary osteoblasts were isolated from calvaria of 5-day-old WT mice following an established protocol^32^. Osteoblasts (5 × 10^3^ cells/well) were seeded in a 96-well plate the day before starting the coculture. Bone marrow cells were suspended in α-MEM containing 10% FBS and 1 × penicillin/streptomycin, 10^−7^ M dexamethasone, and 10^−8^ M 1α- and 25-dihydroxyvitamin D3 and added into each well. In selected wells, WT or mutant hOPG were added to final concentration of 1–100 ng/ml. The medium was replaced every 2 days until the appearance of giant osteoclasts, which usually takes 5–6 days. A Leukocyte Acid Phosphatase kit (Sigma) was used to stain osteoclasts. The TRAP activity was quantified by first lysing cells with 50 µl of lysis buffer (50 mM Tris, pH 7.5, 150 mM NaCl, 1% Nonidet P-40), and incubating 10 µl of lysate with an assay buffer containing 0.5 M sodium acetate, 10 mM tartrate, and 10 mM p-nitrophenyl phosphate substrate for 15 min at 37 °C. The reaction was stopped by adding 0.5 M sodium hydroxide and the absorbance was measured at 405 nm.

*Monoculture Osteoclastogenesis Assay*—Osteoclastogenesis of non-adherent bone marrow cells was induced by adding 20 ng/ml of M-CSF (Biolegend) and 50 ng/ml of soluble RANKL (prepared in house) into the medium. In selected wells, WT or mutant hOPG were added to 25–400 ng/ml.

### Isolation of primary murine articular chondrocytes

Murine articular chondrocytes were isolated from 6-day old pups following an established protocol^33^. Collagen II staining was used to confirm successful isolation of articular chondrocytes. Briefly, chondrocytes were fixed with 4% PFA and stained with mouse anti-type II collagen monoclonal antibody cocktail (CB-11, Chondrex, Inc) at 0.5 µg/ml, followed by Goat-anti-mouse-IgG 649.

### TUNEL staining

Chondrocyte were either untreated or treated with TRAIL at 50–200 ng/ml for 48 h. In selected wells, chondrocytes were co-treated with 100 ng/ml TRAIL and 400 ng/ml WT or OPG^+19^ mutant for 48 h. TUNEL staining was performed using an apoptosis terminal deoxynucleotidyl transferase (TdT) DNA fragment detection kit (ThermoFisher), according to the manufacturer’s instructions. Briefly, cells were fixed with 4% paraformaldehyde in PBS and permeabilized with 0.25% Triton™ X-100. Cells were then incubated with TdT reaction mixture for 60 min at 37 °C, followed by incubation with Click-iT™ Plus TUNEL reaction cocktail (with Alexa647 dye) for 30 min at 37 °C. Slides were mounted with Prolong mounting medium with DAPI and images were taken with a Nikon Ci-S fluorescence microscope. 6 images were used for quantification. The experiment was repeated three times with similar results.

### ELISA assay of hOPG/RANKL binding

Binding affinity of WT and mutant hOPG to RANKL was measured by ELISA. Briefly, 96-well plate was coated by recombinant sRANKL (prepared in the lab)^6^ and blocked by 1% BSA. Recombinant WT and mutant hOPG with concentrations at 1 ng/ml to 1000 ng/ml were added into wells and incubated for 2 h followed by incubation with biotinylated rabbit anti-OPG antibody for 1 h and streptavidin-HRP for 30 min. TMB substrate (Invitrogen) for HRP was used for color development. The absorbance at 450 nm was measured by a plate reader. Apparent Kd value was calculated using Prism software.

### Overexpression of OPG^+19^ in human chondrocyte cell line

TC28a2 chondrocytes (Sigma-Millipore, SCC042) were used for WT OPG and OPG^+19^ transfection. The day before transfection, chondrocytes (passage 3 to 5) were seeded into a 12-well plate at 8 × 10^4^ cells per well to allow cells to reach 60% ~ 80% confluence on the day of transfection. For each well, 1 ug expression plasmid was mixed with 2.5 µl lipofectamine LTX (Invitrogen) in 200 ul Opti-MEM I, and the complex was added onto cells in 800 µl culture medium (DMEM and 10% FBS without antibiotics). 48 h post transfection, medium (1 ml) was harvested and the cell monolayer was lysed with 100 µl RIPA buffer to prepare cell lysate. The concentration of WT OPG and OPG^+19^ was determined by a sandwich ELISA using our rabbit anti-OPG polyclonal antibody as capture antibody, and biotinylated rabbit anti-OPG polyclonal antibody as detection antibody. Standard curves for determining the concentration of WT OPG and OPG^+19^ in the medium and cell lysate was generated using recombinant WT OPG and OPG^+19^ (0.5–200 ng/ml), respectively. To visualize WT OPG and OPG^+19^ in conditioned medium, the medium was purified by a small heparin Sepharose gravity column and after column wash all bound proteins was eluted with 400 µl 1.5 M NaCl in 25 mM HEPES, pH7.1. This step removes most serum proteins from the medium, which contains 10% FBS. 40 µl of the material eluted from heparin column was resolved by SDS-PAGE and blotted with rabbit anti-OPG antibody (Fig. 6A).

### Pyrophosphate (PPi) assay

Extracellular PPi concentration was measured 48 h after human chondrocytes (TC28a2) were transfected with plasmids coding for WT OPG or OPG^+19^ . PPi levels in conditioned media were determined using a non-radioactive bioluminescent assay kit (Lonza). Briefly, 20 µl of converting reagent was mixed with 40 µl of sample and incubated at room temperature for 30 min, after which 20 µl of detection reagent was added and incubated for an additional 30 min. Luminescent signal was measured using a 96 well plate on Flexstation III plate reader (Molecular Devices) according to the manufacturers' recommendations (0.1 s integrated reading). To determine the PPi concentration, a PPi standard curve was generated using sodium pyrophosphate decahydrate (0.02–5 µM).

### Statistical analysis

All data are expressed as means ± SDs. Statistical significance was assessed using two-tailed Student’s t-tests or analysis of variance (ANOVA) using GraphPad Prism software (GraphPad Software Inc.). *p* value < 0.05 was considered significant.

### Ethical approval

All animal works in this study have been approved by the institutional animal care and use committee of the University at Buffalo (protocol number: ORB14126N), and the study is reported in accordance with ARRIVE guidelines. All methods were performed in accordance with the relevant guidelines and regulations.

## Data Availability

The authors declare that all data supporting the findings of this study are available within the article.
